# No evidence for kin recognition in a passerine bird

**DOI:** 10.1371/journal.pone.0213486

**Published:** 2019-10-23

**Authors:** Martina Lattore, Shinichi Nakagawa, Terry Burke, Mireia Plaza, Julia Schroeder

**Affiliations:** 1 Department of Life Science, Imperial College London, Silwood Park, Ascot, United Kingdom; 2 Evolution & Ecology Research Centre and School of Biological, Earth and Environmental Sciences, University of New South Wales, Sydney, Australia; 3 Department of Animal and Plant Sciences, University of Sheffield, Sheffield, United Kingdom; 4 Department of Evolutionary Ecology, National Museum of Natural Sciencie-CSIC, Madrid, Spain; Universidade de São paulo, BRAZIL

## Abstract

Theory predicts that individuals behave altruistically towards their relatives. Hence, some form of kin recognition is useful for individuals to optimize their behavior. In species that display bi-parental care and are subject to extra-pair matings, kin recognition theoretically can allow cuckolded fathers to reduce their parental investment, and thus optimize their fitness. Whether this is possible remains unclear in birds. This study investigates whether males provide differential parental care depending on relatedness, as a proxy to recognizing chicks in their nest as kin or not. We cross-fostered House sparrow (*Passer domesticus*) chicks after hatching, and then expected that fathers would show a decrease in their parental efforts when tending to a clutch of unrelated offspring. House sparrow males are able to adjust their parental care to the identity of their partner, making them an ideal study species. However, there was no significant effect of relatedness on provisioning rates. This suggests that sparrows may not be capable of kin recognition, or at least do not display kin discrimination despite its apparent evolutionary advantage.

## Introduction

Kin recognition is the ability to recognize the degree of relatedness with other individuals [1] and evidence for it has been found to be widespread across many taxa [2, 3, 4, 5]. Indeed, individuals can behave altruistically [6] and more so the more two individuals are related [7]. Hence relatedness is a determining factor in understanding how altruism works in nature, and therefore a driver of behavioral evolution [8].

Relatedness is the proportion of shared genes between conspecifics. Building upon Fisher’s [9] work on altruistic behavior, Hamilton [7] postulated that an altruistic behavior will be expressed when the costs of the behavior are lower than the fitness benefits to the individual benefitting from the altruistic behavior, modulated by the degree of relatedness. According to Hamilton’s Rule, for a behavior to be considered altruistic the following equation must be achieved:

rb–c>0

where *r* is the genetic relatedness between individuals, b is the fitness benefit to the receiver, c is the fitness cost to the altruist. Hence, *rb* corresponds to the indirect fitness effects of a trait, whereas -c is the direct effect [10]. For instance, in a parents-offspring relationship, the parent incurs costs, by spending energy finding food, not eating it themselves but feeding it to their young. Yet, they are genetically related to their offspring, and thus the fitness benefit that the offspring received by being fed translates into fitness for the parents–but only as long as they are related to each other. Hence, mechanisms allowing individuals to recognize relatives may be useful, in species where kin-selection is favorable [7].

There is an ongoing debate on the details of the definition of kin recognition, considering different aspects of it, including genetic and environment-based cues and mechanisms. For the purposes of this work, the most relevant definition is the operational one suggested by Holmes and Sherman [11], with kin recognition being defined as the “differential treatment of conspecifics differing in genetic relatedness” (also referred to as “kin discrimination” [1]).

There are numerous mechanisms for individuals to recognize their genetic relatives. Contextual cues include spatial and temporal factors, and this mechanism is quite common among animal species [8]. Birds, in particular, often seem to recognize any chick in their nest as their own, however they can also take into account their access to a mate and the probability of their paternity of the brood to judge whether the young is theirs [12]. Instead, kin recognition via phenotypic cues takes into consideration a variety of phenotypic traits of the individuals. The “prior association hypothesis” is based on direct familiarity, where individuals recognize their own kin by first becoming familiar with their phenotypes in a shared, early environment and then recognizing them in the future [8, 11]. The “phenotype matching hypothesis” suggests that “individuals who resemble their own kin are treated as related” [8].

The ability to recognize their own kin can enable individuals to adjust their behavior according to the degree of relatedness to others [1, 11]. In species that display biparental care such as many social birds, it is thought the father’s effort might be affected by his relatedness to the nestlings if he is capable of recognizing them [13]. It has been previously observed that providing food and care to offspring involves significant opportunity costs to male birds, in the form of reduced self-maintenance (by providing food to the young instead of themselves), risks of predation due to more numerous foraging trips [14]. These costs are especially important in birds that mate and reproduce multiple times during a year and during their lifetime, as they significantly reduce their chances of reproducing again due to the energy and time they spend that decreases their chance of mating again in a short span of time [15]. Caring for unrelated offspring does not improve an individual’s fitness as there are only costs to raising offspring that does not share any of their genetic material [16]. Thus, it is best avoided or reduced [16]. Females are typically sure of their genetic relatedness to their young, but fathers experience a greater uncertainty of paternity, and thus one would expect it to be beneficial if fathers could distinguish between kin and non-kin, and then reduce their parental care to young they are not related to.

Some experimental and observational research on kin recognition has produced contrasting results [17, 18, 19, 20], due to different risks of extra-pair offspring and other ecological factors in the studies. For example, Boncoraglio and Saino [19] showed that Barn Swallows (*Hirundo rustica*) fathers do not underfeed unrelated chicks, even though chicks themselves beg differently when in mixed broods. In contrast, Zebra Finches (*Taeniopygia guttata*) appear to behave differently towards related and unrelated offspring [20].

Because of this limited knowledge on the mechanism of kin recognition in birds, we here test the hypothesis that males are capable of kin recognition and adjust parental care accordingly, in a large, long-term dataset with information on genetic relatedness in House Sparrows (*Passer domesticus*). This social bird species has a relatively high extra-pair paternity rate [21, 22], a relatively stable social mating system, and we know that male house sparrows are able to adjust their parental care to social cues [23]. These traits make house sparrows an ideal model to investigate this hypothesis. While extra-pair matings can be beneficial for the extra-pair sires as they gain more offspring while not being required to spend energy by attending to them, female infidelity can have negative effects for her own fitness [24, 25], and that of the within-pair mates, as males receive no benefit from caring for chicks that do not share their genes [16]. We therefore expect males to provide less parental care to clutches that include unrelated nestlings. This requires the males to be able to recognize their own kin to adjust their level of care.

## Methods

This study did not directly require an ethical board to approve the protocol, as the data was collected previously as part of the long-running Lundy Sparrow Project. The Lundy Sparrow Project operated under PPL 40/3521 granted to Terry Burke, and PL 7008082 to Julia Schroeder by the Secretary of State of the UK Home Office.

### Study population

This research was carried out on a population of house sparrows that has been monitored since 2000 on Lundy Island, 19km off the coast of Devon in the Bristol Channel (51°10′N, 4°40′W), where levels of migration reach a maximum of three birds every four years [22], making it a nearly closed population suitable for longitudinal studies. Life histories of individual birds and a full pedigree of the population are available, therefore the identities of parents for each brood are known [22]. Birds are individually marked with a unique combination of three colored rings and a numbered metal ring [26]. In addition, each sparrow was provided with a subcutaneous passive integrated transponder which is read by radio frequency identification (RFID) antennas attached to each nest-box on the island [27]. Of all breeding attempts, 95% occurs within these nest-boxes, which were systematically monitored both by direct observations and using cameras as follows [26].

The house sparrow is a social bird with bi-parental care. Adults form social pairs that last for one or multiple breeding seasons, and sparrows have multiple broods per year [28]. However, while socially monogamous, there is some genetic infidelity present in this species [22]. Previous studies on this population indicated that 37.9% of all the broods in the population had at least one extra-pair chick, with 17.5% of all the offspring born during the years of observation being extra-pair [24]. The opportunity costs of providing care to unrelated offspring are particularly relevant in such a species that reproduces numerous time over the years, as spending unnecessary energy can lead to reduced chances to breed in the future [28]. Furthermore, males seem to employ a bet-hedging strategy where they adjust their parental care according to the female they are paired with, and while there is a genetic basis, most of the within-male variation is due to phenotypic plasticity or unexplained [14, 27]. Thus it is a suitable species to study kin recognition between parents and offspring.

### Data collection

We used data collected from chicks born between 2004 and 2015, as part of the Lundy Island Sparrow Project. The chicks were cross-fostered on the day after hatching and remained in their rearing brood until fledging. Each brood underwent one of three treatments, where the brood was either not cross-fostered, partially cross-fostered, or fully cross-fostered [29]. However, we had information about genetic paternity in all broods, and we used that to consider the status of the father in relation to the brood it attended. Using all this information, we created a variable with three treatment levels with respect to the genetic relatedness to the social father who attended the brood: fully related (-1); partially related (0); unrelated (1).

Every single brood was given an individual identifier (BroodID), and then all were monitored with video-cameras placed between 2 and 5 meters away from their entrance, with a field of view of 30cm around the nest-box [26]. Videos were recorded in the morning between day 1 and day 15 after hatching. This study uses the time from when a bird is first seen on video to the end of the recording as “effective video time”, to account for the fact that individuals could need time to adjust to presence of a camera and therefore take longer to return to the nests at first [26]. Effective video times range between 10 and 120.26 minutes, with a mean of 88.34 minutes. By watching the videos we extracted the number of parents visits to the nest and we calculated the provisioning rate for each parent (number of visits per hour). Only the provisioning rate of the social father was used in this research.

### Statistical analysis

We analyzed the data with R [30]. We ran a linear mixed effects model to test the hypothesis that male birds reduce their provisioning rate when attending to broods that hold chicks they are not related to. In this model we used the number of provisioning visits per hour by the attending male as the response variable. Whether or not a clutch was related to the care-providing father was used as explanatory variables. The standardized age of chicks at the time of the video and the number of hatchlings in a nest were used as additional fixed covariates, as we know they are related to parental care [23]. We also included the brood ID as additional random effect in order to account for the repeated measurements in broods over several days. We used the within-subject centering method [31] to distinguish between a bias of cross-fostered nests towards nests with attending males with higher or lower parental care, and *vice versa*. Such a bias could come across if cross-fostering cannot always happen completely randomly [29], due to cross-fostering always requiring at least two broods of the same age. This means that by definition, broods that are less synchronized with others, such as the very early or very late broods, are unlikely to be cross-fostered. The parents tending to these nests may be systematically different from those that breed during time periods when most of the population breeds, as early birds may be of higher quality, and late birds may be of lower quality, on average. To distinguish between a male displaying different parental care towards broods with young that are related to him *versus* a brood with young unrelated to him, and differences in parental care between different male individuals that care for broods that are cross-fostered, and males that care for broods that are not cross-fostered, we created two new fixed covariates from the variable treatment (Tb and Tw) (-1 = genetic sire to all offspring in the nest, 0 = attending male is related to part of the offspring in the nest, either through partial cross-fostering or through extra-pair paternity, 1 = attending male is unrelated to the offspring in the nest through full cross-fostering). To estimate and capture the between-male variation, we calculated the average value for each male of the treatment. Thus, if a male twice tended to a fully cross-fostered brood (both with a value of 1), and once for a brood containing only his genetic offspring (with a value of -1), this between-male treatment effect (T_b_ in the following) would be 0.33 for each of these observations. To estimate the within-male variation, we subtracted the mean treatment of each male from the treatment value of each observation of every respective individual male. Thus, the male from above would get a value of 0.67 for the first two broods, and -1.33 for the second one for the within-male treatment effect (T_w_ in the following). We used the attending male’s ID as a random effect on the intercept.

The model was run in MCMCglmm (Hadfield 2010). We present parameter estimates with their respective 95% credible intervals (95CI). We considered fixed effects as statistically significant if their 95CIs did not span 0. We also present the pMCMC, that is approximately twice the MCMC estimate of the probability that the 95CI does not span 0, a value analogue to traditional *P* values.

## Results

We used 2388 video observations collected between 2004 and 2015. We extracted parental care rate for the male from each video, which were from 296 individual male sparrows. Only 19 males were observed only once, all other males were observed repeatedly (Table 1). The median number of times a male was observed was 5, the mean was 7.98 times. Our data was also structured by brood. Of the 1048 broods, 164 broods were only observed once, with an overall median of each brood being observed two times (Table 1).

**Table 1:** The frequencies of individual repeated observations of parental care of Lundy sparrow males tending to their broods between 2004 and 2015.

	Frequencies	
Repeats	Individual males	Individual broods
1	19	164
2	51	584
3	20	216
4	39	72
5–10	100	9
11–20	43	3
21–30	18	0
31–40	4	0
41–50	2	0

**Fig 1:**
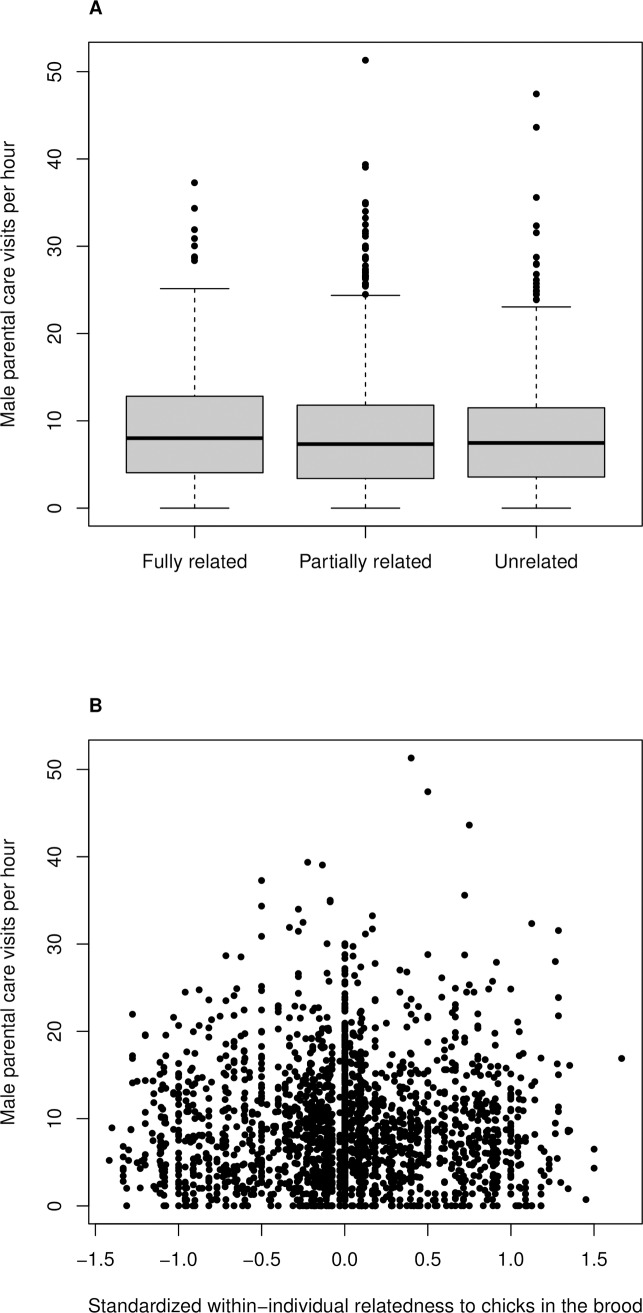
A Male parental care per hour in relation to his relatedness to the brood he cares for. B Male parental care in relation to the difference between an observation and the average relatedness of the respective male to the chicks he cares for. This standardized variable represents variation within individuals. Larger values represent a lower relatedness between the male and his brood. The hinges represent the first and third quartiles, the thick line is the mean, and the dots represent single observations outside of the hinges.

Of all 1048 broods, 394 were from non-cross-fostered broods, 196 from fully cross-fostered broods and 458 from partially cross-fostered broods. After accounting for the genetic relatedness of the offspring present in the nest in relation to the attending male, we had 227 broods attended by a male who was related to all offspring in the brood, 591 broods in which some of the chicks were related to the attending male, and 230 broods where the male was completely unrelated to the chicks he was caring for. Males tended to attend to broods completely related to them with 8.9±0.3 (mean ± SE) visits per hour on average, to partially related broods with 8.8±0.3 visits per hour, and to completely unrelated broods with 8.6±0.4 visits per hour (Fig 1).

The analysis showed that males did not change their parental care when caring for a brood containing chicks that were not genetically related to them (Table 2).

**Table 2:** Results from a linear mixed model. Chick age and the number of hatchlings were standardized to a mean of 0 and variance of 1. The treatment refers to the degree of which the chicks were related to the attending male (low = high relatedness, high = low relatedness). N = 2388 observations of parental care between 2004 and 2015, on 1048 broods and 299 individual males.

Parameter	Estimate	Precision	Probability
*Fixed effects*	*B*	*95CI*	*pMCMC*
Intercept	8.80	8.37–9.25	<0.001
Within-individual treatment	0.19	-0.37–0.75	0.50
Between-individual treatment	-0.86	-1.18–0.16	0.10
Chick Age	0.32	0.11–0.54	0.002
Number of hatchlings	0.38	0.09–0.68	0.01
*Random Effects*	*Variance*	*95CI*	
Brood reference	12.74	9.97–15.47	
Male ID	6.07	3.65–8.46	
Residual	24.85	22.92–26.76	

## Discussion

We found no support for the idea that male house sparrows adjust paternal care according to relatedness, which suggests there is no kin recognition. Little research has previously been done on kin recognition and paternal care in house sparrows [23]. House sparrows have been shown in the past to be capable of paternal care adjustment, as males with cheating partners show reduced parental care investment [23]. While no support was found for between-male effects, Schroeder et *al*. identified a significant within-male effect. This showed individual sparrows adapted their parental investment depending on the level of their female’s fidelity and there was a significant adjustment in paternal care when the males changed partners [23]. Here, we specifically investigated whether this adjustment was due to the amount of unrelated offspring being present at the nest. If an adult male was able to recognize its own chicks, we would expect to see different behavior at nests where we cross-fostered the chicks and where we did not.

This study shows that males do not decrease their provisioning rate towards cross-fostered (or otherwise unrelated) chicks, despite the apparent evolutionary advantage of paternal care adjustment to unrelated offspring. Therefore, this experimental result, with a high statistical power, does not support the idea that male sparrows are able of kin recognition. While our study did not conclusively rule out kin recognition (males could recognize their kin but not react to this information), it is, to our best knowledge, the largest experimental dataset in the wild that is currently available and the first to have specifically manipulated conditions for paternal care adjustment in relation to kin recognition before [18, 32, 33].

It has previously been suggested that trade-offs might limit the advantage of kin discrimination. As one can imagine that kin recognition might not be perfect, the balance between the costs of wrongly rejecting offspring and the costs of wrongful acceptance will play an important role [8, 13, 19, 23]. Furthermore, only part of the chicks in a brood may be unrelated, while another part is related, and a reduction in parental care may be difficult to target at certain chicks only. Moreover, if females seek extra-pair matings to improve their reproductive output, especially by increasing the quality of the genes of their offspring, they would have interest in their extra-pair offspring growing [13]. Therefore, if the males were to reduce their parental investment, females might increase their own investment. The interplay between male and female parental care is complex, as it is guided by sexual conflict, cooperation, compatibility and several phenotypic, and environmental, indirect social effects [24].

Another explanation for our findings is that kin recognition might derive from learned cues, which require longer to develop than the two days parents spent with their own chicks in this experiment before cross-fostering [32]. For example, Razorbills (*Alca torda*) accept unrelated chicks in their nest and raise them without distinctions within the first 15 days of their lives, whereas if foreign chicks are introduced after 16 days they get rejected [34]. The same phenomenon was observed in European Starlings (*Sturnus vulgaris*), with foreign chicks over 16 days old not being accepted into unrelated broods [35]. The sparrows on Lundy island were a maximum of two days old when cross-fostered (because sometimes not all eggs hatch on the same day), which might have affected the fathers’ ability to recognize them, not being given time to learn their phenotypic cues. If this was the case, it would suggest kin recognition occurs by prior association [8, 11] Future studies using cross-fostering of chicks at a later age could be useful to investigate this.

In summary, this long-term experiment, providing a large amount of data, did not provide support for kin discrimination in the house sparrow and suggests that they are not capable of kin recognition.

## Supporting information

S1 File Randomized data. This is the randomised data necessary to reproduce the experiment.
(CSV)
